# 3D Reconstruction of the Human Airway Mucosa *In Vitro* as an Experimental Model to Study NTHi Infections

**DOI:** 10.1371/journal.pone.0153985

**Published:** 2016-04-21

**Authors:** Pasquale Marrazzo, Silvia Maccari, Annarita Taddei, Luke Bevan, John Telford, Marco Soriani, Alfredo Pezzicoli

**Affiliations:** 1 GSK Vaccines S.r.l., via Fiorentina 1, 53100, Siena, Italy; 2 Interdepartmental Centre for Electron Microscopy, Tuscia University, Viterbo, Italy; 3 Respiratory Disease Area, Novartis Institutes for BioMedical Research, Horsham, RH12 5AB, United Kingdom; Louisiana State University, UNITED STATES

## Abstract

We have established an *in vitro* 3D system which recapitulates the human tracheo-bronchial mucosa comprehensive of the pseudostratified epithelium and the underlying stromal tissue. In particular, we reported that the mature model, entirely constituted of primary cells of human origin, develops key markers proper of the native tissue such as the mucociliary differentiation of the epithelial sheet and the formation of the basement membrane. The infection of the pseudo-tissue with a strain of NonTypeable *Haemophilus influenzae* results in bacteria association and crossing of the mucus layer leading to an apparent targeting of the stromal space where they release large amounts of vesicles and form macro-structures. In summary, we propose our *in vitro* model as a reliable and potentially customizable system to study mid/long term host-pathogen processes.

## Introduction

In the last decades, the exploitation of transformed and/or immortalized cell line monocultures turned out to be a powerful approach not only to unravel the mechanisms of infection for various microbial pathogens, but to allow the characterization of thousands of drug compounds. Despite their proved utility, the need for more accurate and physiological systems has driven researchers to develop models based on two or more cellular lineages, including epithelial and immune cells [1]. Recently, to develop structured 3D models, scientists have engineered biocompatible scaffolds and bioreactor-based culture systems that have brought the biological relevance of these models to a very high level. For example, supporting scaffolds and biomaterials provide the framework in which cells can deposit extracellular matrix components and differentiate to form a functionally relevant tissue. In this context the use of different cellular types allows a nearly exact reproduction of human specific anatomical districts that are of exceptional value if one wants to follow host-pathogen interaction phenomena [2, 3]. The complexity of these systems not only allows the characterization of basic interactions such as bacterial adhesion or internalization patterns, but could also potentially open new perspective on the study of elaborate bacterial-host interaction phenomena, such as the assessment of immune clearance mechanisms within the mucosal environment and the long-term characterization of microbial persistence strategies. One of the major concerns about the use of animal models to study bacterial infectiveness is that the vast majority of pathogens have a strict specificity for their host. Likewise, the use of human tissue explants is confined to a limited number of models due to the complex cyto-architecture that hinders the circulation of nutrients leading to the deterioration of the sample within few days [4, 5]. Therefore, in some cases, assembling physiological *in vitro* systems that faithfully reproduce the native tissue represents a valid alternative to the use of animals or human explant cultures. A number of models reconstituting the human respiratory mucosa *in vitro* have been successfully established during the last years. Frequently these models comprise the co-culture of fibroblasts and bronchial epithelial cells that are assembled on biocompatible scaffolds or porous membranes. Fibroblasts have a positive effect on the epithelial cell function by increasing proliferation, guiding the differentiation, modulating mucin secretion and inducing a correct spatial distribution [6–8]. These events contribute to an appropriate assembling of the bronchial epithelium and, by establishing a spatially defined structure, to the maintenance of the mucociliary phenotype for a long period [9]. In recent years, a number of strategies have been proposed to optimally embed fibroblasts in 3D airway models [10–13]. Nevertheless, Pageau and colleagues have shown that the source of fibroblasts is critical to the differentiation of the epithelial cells [8].

Dual or triple co-culture models implanting immune system components in pseudo-tissues, have also been used to characterize immune defense mechanisms and elucidate the paracrine signaling of cytokines on the epithelium [2]. In particular immune-competent *in vitro* 3D models of the airways have been used to characterize the response to allergens or foreign particles exposure and the human dendritic cell function within the lung environment [14–16]. Of interest, models of the airway wall providing the use of mesenchymal stem cells (MSCs) in combination with epithelial cells have been exploited for the characterization of regenerative and wound repair mechanisms [17, 18].

The aim of our study is to recreate a physiological model mimicking the human tracheo-bronchial mucosa, including the epithelium and the supporting stromal tissue. The use of a porous inert scaffold provided the mechanical support and the *in vivo*-like scale for co-culturing the cells forming the basic system (named BEM, acronym of Bronchial Epithelial Model), constituted by human normal fibroblasts and epithelial cells. The characterization of the BEM highlighted the correct formation of the basal membrane (BM) and the presence of a sustained mucociliary differentiation of the epithelium. The exploitation of the proposed model to monitor nontypeable *Haemophilus influenzae* (NTHi) infections, by confirming the invasive phenotype observed in human explants supports the use of this reconstituted mucosal pseudo-tissue for a number of pharmaceutical and medical applications.

## Results

### The bronchial equivalent model strictly resembles the human respiratory mucosa

**Fig 1:**
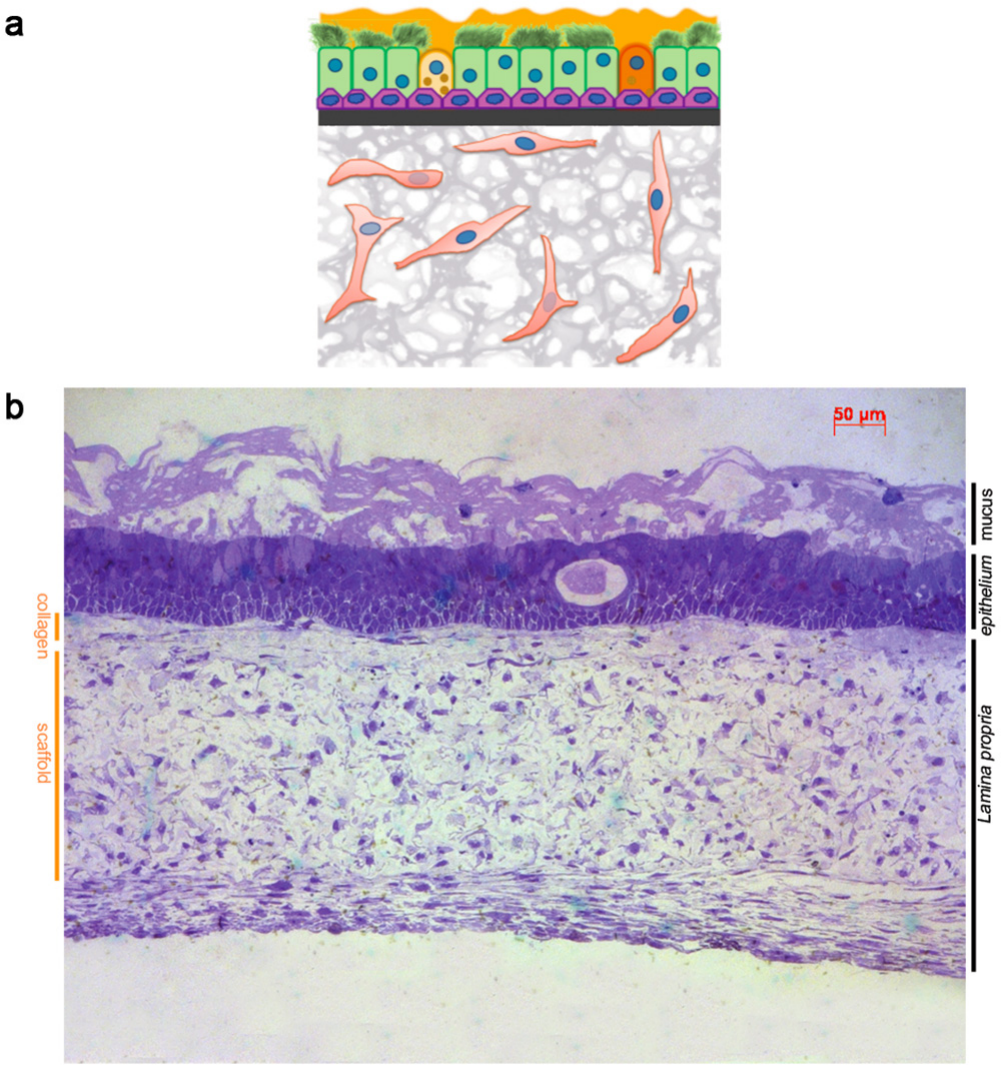
Schematic representation of the bronchial equivalent model. a) Schematic representation of the basic BEM, consisting of NHLFs and NHBE cells. b) Toluidine blue staining of a mature standard BEM.

**Fig 2:**
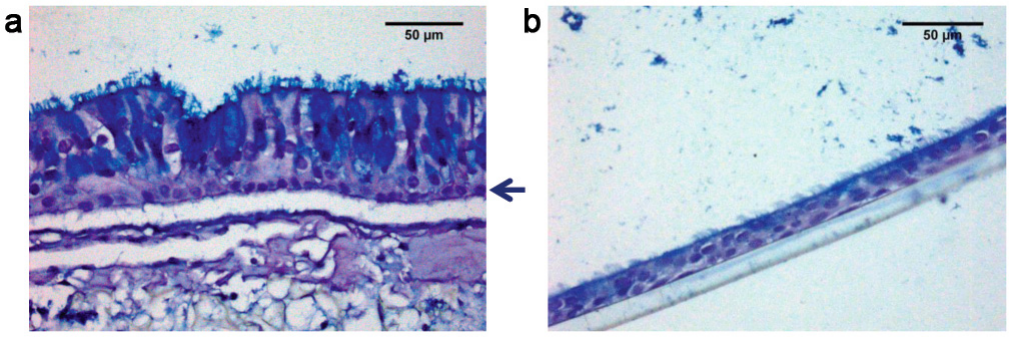
Different morphology of NHBE cells grown on BEM or transwell. Haematoxylin-eosin-alcian blue staining of NHBE cells differentiated on a BEM (a) or on a transwell system (b). The arrow indicates the basal layer that forms when NHBE cells are grown on a BEM. Images are representative of 4 different experiments.

**Fig 3:**
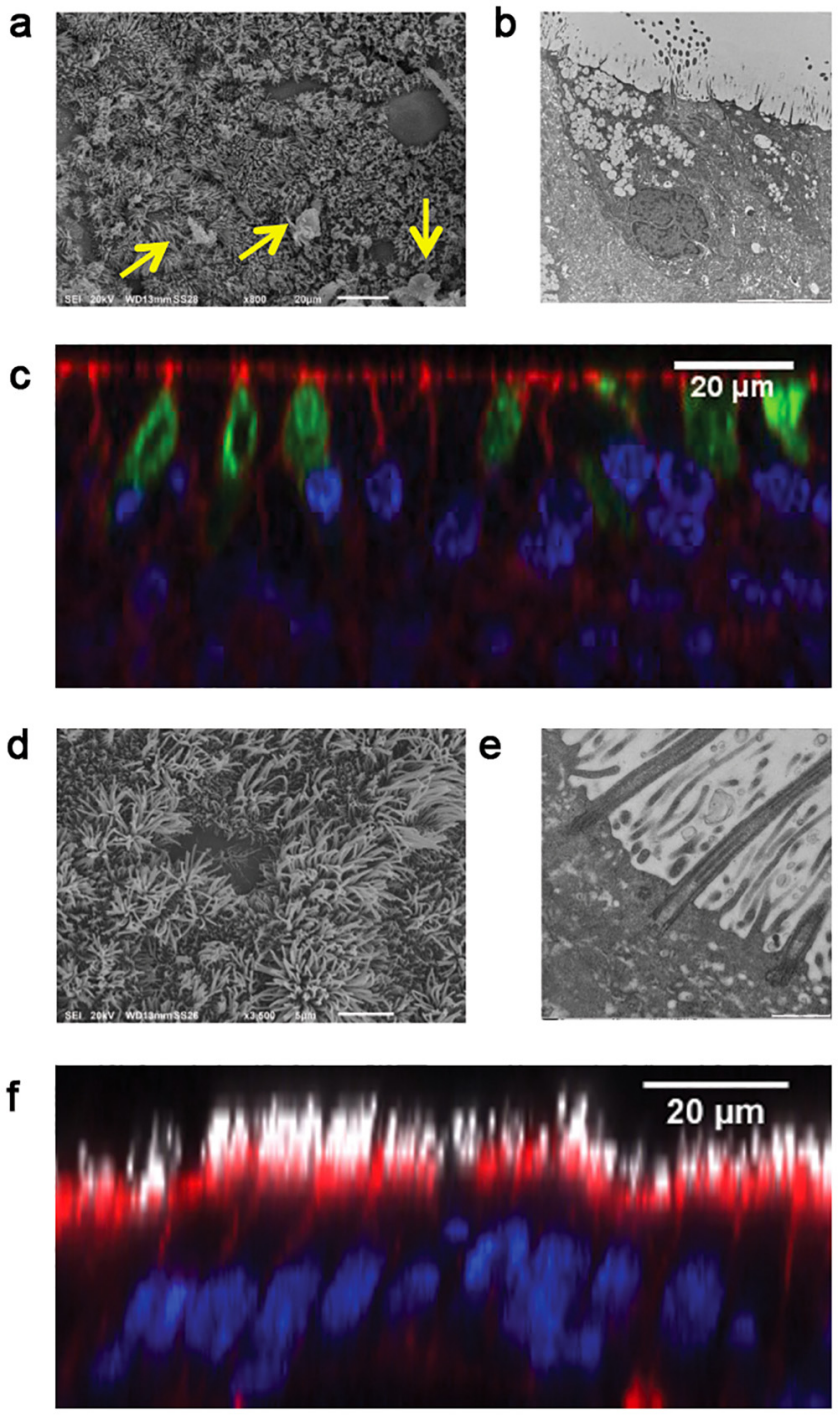
Mucociliary differentiation of NHBE cells grown on a BEM. Mucus granules are visible by SEM (a) (indicated by arrows), TEM (b) and confocal analysis (c) (blue is DNA, green is MUC5AC, red is F-actin). Cilia are visible by SEM (d), TEM (e) and confocal analysis (f) (blue is DNA, white is B-tubulin, red is F-actin). Images are representative of 3 independent experiments.

With the aim of reconstituting a human bronchial equivalent model, we co-cultured human-derived primary cells isolated from healthy individuals and spatially organized on a porous inert scaffold (see Materials and Methods). We firstly seeded the scaffold with fibroblasts (NHLFs) and cultured to allow fibroblasts to deposit ECM components such as fibronectin and collagen (S1 Fig). Subsequently a layer of rat tail collagen I was poured on top of the scaffold to offer support to the epithelial sheet constituted of primary human tracheo-bronchial cells (NHBE). The obtained pseudo-tissue (Fig 1A) was finally cultured at air-liquid interface (ALI) for three weeks to allow the full differentiation of the epithelial sheet. The characterization of the BEM was based on the analysis of 4 independent experiments, each consisting in the assembly of more than 3 replicates. In order to exclude that the results could be subject-bound, we used cells from different human sources during the first two experiments with respect to the last two. The mature BEM was typically 300 microns thick and highly resembled the human respiratory mucosa. Indeed, as shown in Fig 1B, toluidine blue staining of BEM sections revealed the presence of a well-formed columnar epithelium typical of the native tissue. The formation of a thick mucus film covering the epithelium was also evident. We observed that the thickness of the formed mucus layer could be influenced by culture conditions, such as ALI phase duration, stromal cellular density and composition and/or washing frequency of the apical surface to simulate the physiological displacement of mucus substances (data not shown). Differences in epithelial cell profiles were observed when the BEM was compared with classical transwell system (Fig 2). Indeed, NHBE cells grown under 3D conditions develop an elongated columnar morphology (Fig 2A) while when cultured on transwells usually have a flat cuboidal shape (Fig 2B). Moreover, the BEM shows a well-defined line of nuclei just above the collagen sheet suggesting the formation of a basal layer typical of the human pluristratified mucosal epithelium. Generally the BEM had an increased rate of mucus accumulation, as a consequence of a higher number of mucus-secreting cells with respect to NHBEs cultured on transwell membranes (data not shown). The mucociliary escalator is essential for the respiratory tract physiology and requires the participation of both ciliated (CCs) and goblet cells (GCs) [19, 20]. Scanning electron microscopy (SEM) analysis of the BEM indicated a high-grade of mucinous differentiation (Fig 3a) and a well-distributed carpet of cilia covering the apical surface of the epithelium (Fig 3d). The presence of mucus-producing GCs was confirmed by both TEM analysis and MUC5AC-specific fluorescent immunostaining (Fig 3b and 3c). Outstretched cilia protruded from the basal bodies to the extracellular space and displayed the typical length described for *in vivo* tissues (~7um)[21] (Fig 3e and 3f).

### The BEM forms a basement membrane and constitutes sealing junctions

**Fig 4:**
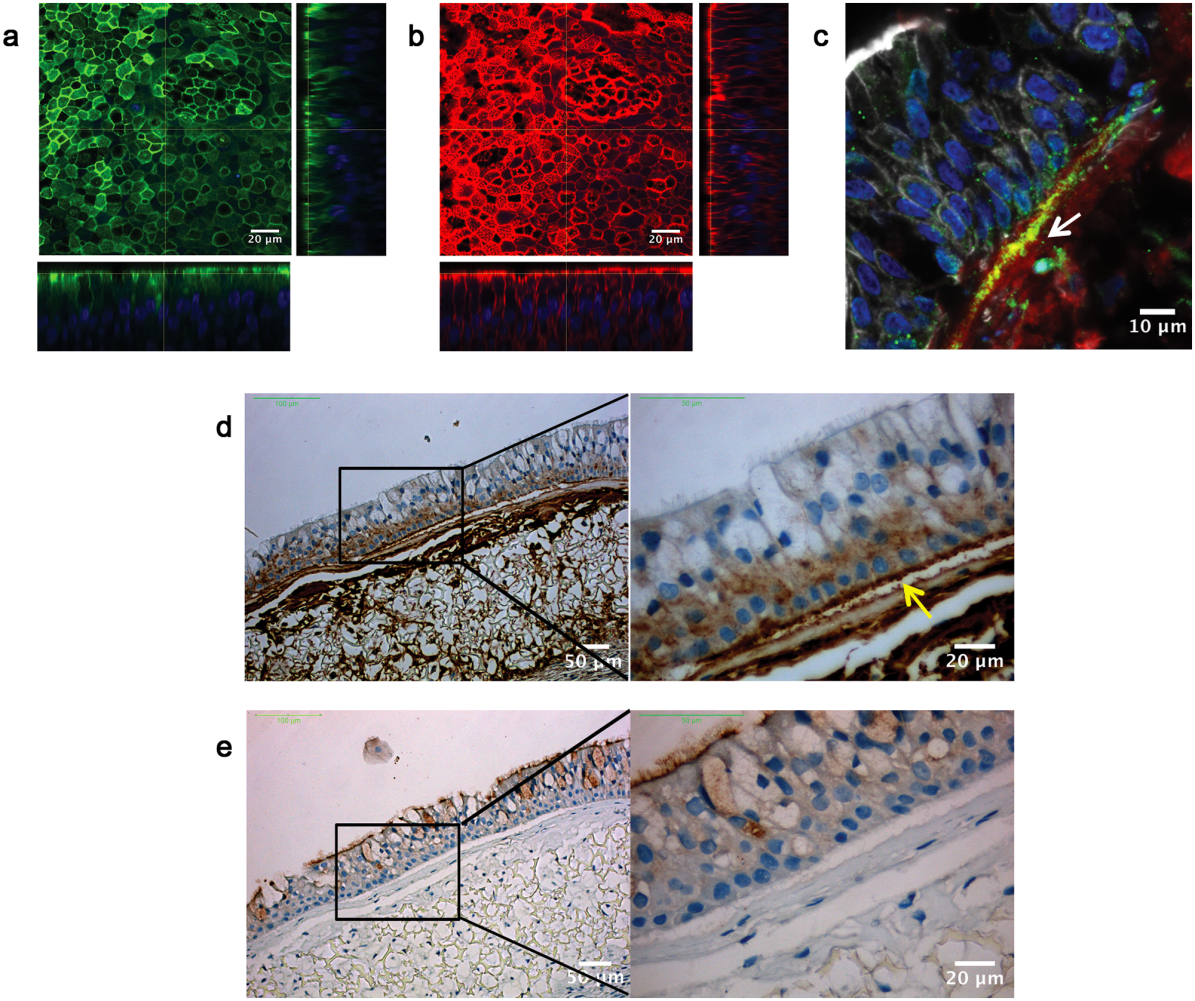
Characterization of differentiation markers of the BEM epithelial barrier. Immunofluorescence staining for a) ZO-1 (green, blue is DNA) and b) F-actin (red). c) Immunofluorescence staining of laminin and integrin alpha-6 (ITGA6) (blue is DNA; red is laminin; green is ITGA6). d) IHC staining of laminin. The arrow indicates the presence of a continuous layer of laminin at the interface between the epithelial sheet and the stromal compartment; antibody isotype control is represented in (e). Images are representative of 4 different experiments.

The epithelium is a physical barrier against pathogens colonizing the human respiratory tract and its integrity is a key requirement. The functionality of a physiological epithelium typically relies on the establishment of tight junctions (TJs) that seal together the epithelial cells forming the barrier [22]. Zonula occludens 1 (ZO-1) protein is an essential component of TJs and, in our BEM, ZO-1 properly delineated inter-cellular contour at the apical side of the NHBE layer (Fig 4a and 4b). *In vivo* epithelial cell polarity is controlled by the basement membrane (BM), a physical extracellular component preserving also host defense capacity [23]. Major components of the BM are laminins that form a network to which epithelial cells can anchor through hemidesmosomes [24]. Immunostaining on BEM cross-sections revealed a notable deposition of laminin (Fig 4d) and also the distinctive presence of the integrin alpha-6 (ITGA6) hemidesmosomal component. Accordingly, both markers co-localized with the collagen layer that separates the epithelial and stromal regions of the BEM (Fig 4c). These data support the evidence that epithelial and fibroblast respiratory cells may synergistically reproduce the formation of the BM *in vitro*.

### The bronchial model develops features associated with epithelial homeostasis and repair function

**Fig 5:**
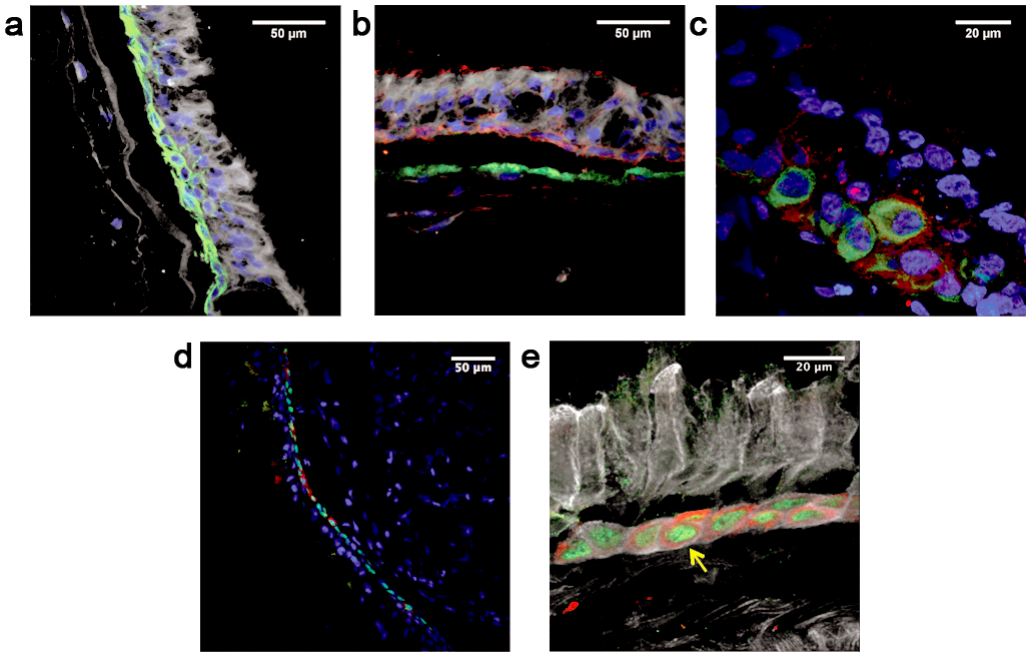
Characterization of epithelial markers by confocal microscopy. a) Immunofluorescence staining of cytokeratin 5 (blue is DNA; green is CK5; white is transmitted light). b) Immunofluorescence staining of nerve growth factor receptor and integrin alpha-6 (blue is DNA; red is NGFR; green is ITGA6; white is transmitted light). c) Immunofluorescence double staining for cytokeratin 5 and nerve growth factor receptor (blue is DNA; red is NGFR; green is CK5). d) Differential expression of basal cells CK14 and p63 markers (blue is DNA; red is CK14; green is p63). e) Enlargement of an area represented in d); the yellow arrow indicates positivity for both p63 and CK14. Images are representative of 4 different experiments.

**Fig 6:**
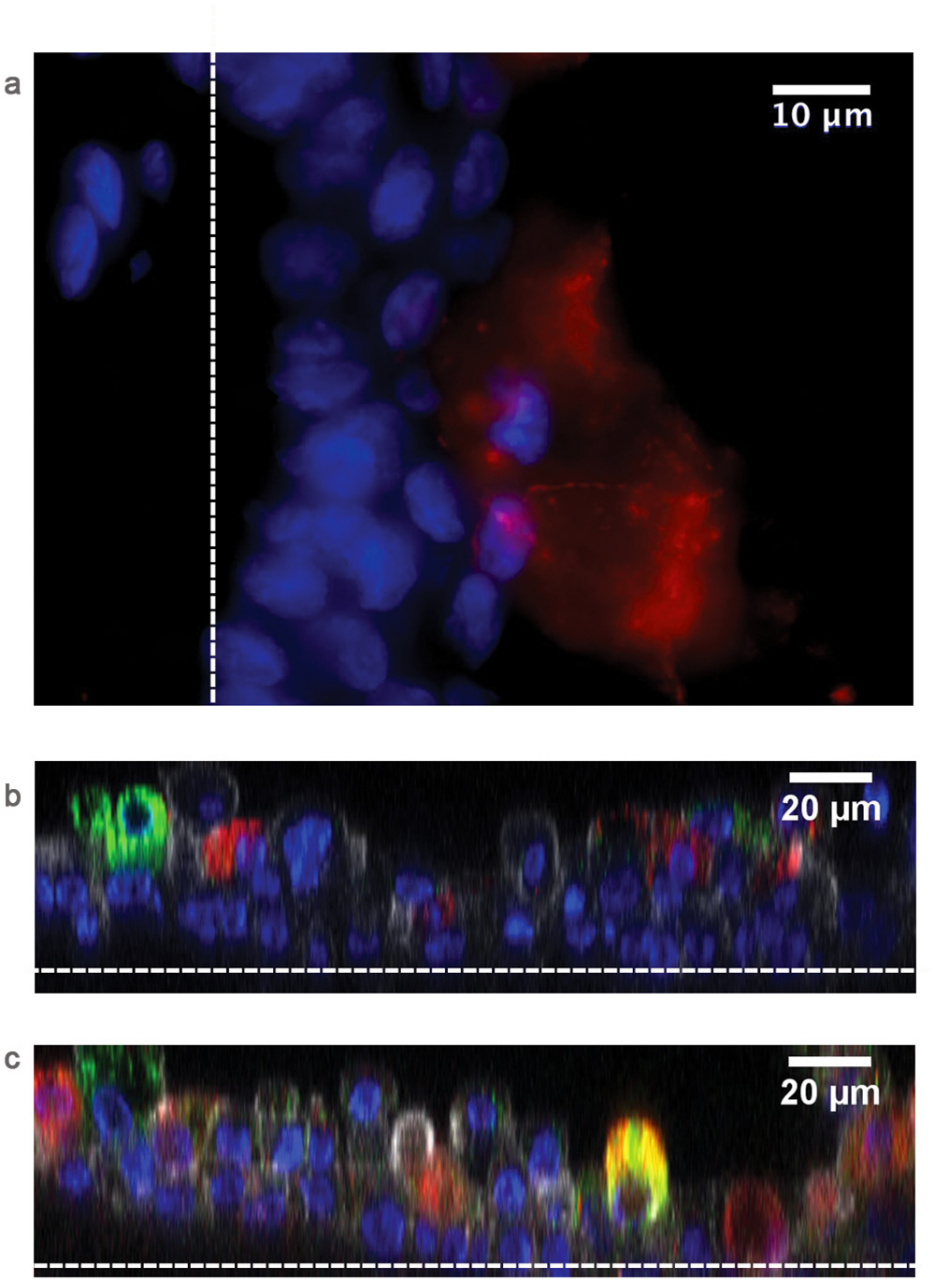
Club and goblet secretory cells are both present in the epithelial space of the BEM. a) Immunofluorescence staining of ClCs (positive for CCSP, red). b) Immunofluorescence staining of ClCs (positive for CCSP, red) and goblet cells (positive for MUC5AC, green). c) Immunofluorescence staining reveals the presence of cells positive for both Club cells and goblet cells markers (blue is DNA; red is CCSP; green is MUC5AC). White dotted lines separate the epithelium from the scaffold. Images are representative of 4 different experiments.

The respiratory system possesses homeostatic mechanisms and a facultative regenerative capacity upon injury that are in charge of different multi-potent progenitors cells, such as basal (BCs) and Club cells (ClCs) [25]. Interestingly the cell layer oriented along the reconstituted BM expressed BC-specific markers as shown in BEM cryosections. Indeed markers as cytokeratin-5 (CK5), nerve growth factor receptor (NGFR) and p63, constitutively present in human airway epithelia, almost uninterruptedly delineated the basal layer of the BEM (Fig 5A–5C). In addition, as shown in Fig 5d and 5e, we found that a minor subset of the p63^+^ cell pool was also positive for CK14 staining. Since CK14 has been described to be upregulated in airways during regeneration after damage [26], the presence of this marker could imply a possible physiological cellular turnover within the BEM epithelium. Altogether these results indicate consistent BC presence in the BEM in line with what has been reported for human respiratory mucosae [27]. Notably, we were able to detect the presence of ClCs within the upper epithelial layer of the differentiated epithelium, as detected by fluorescent staining specific for uteroglobin (also known as CCSP) (Fig 6a). Finally, dual immunofluorescence staining highlighted not only a balance between GCs and ClCs, but also the existence of rare double positive CCSP/MUC5AC population, hypothesizing a linkage between the two strict secretory phenotypes (Fig 6b and 6c).

### Cytokine production is augmented during differentiation time of BEM

In order to define a baseline level for cell signaling molecule secretion by the standard BEM, we monitored the production of a panel of 27 cytokines, by using a Luminex bead-based assay (for experimental details see Materials and Methods). This panel included the main mediators of the inflammatory state, various chemotactic factors and several growth factors indicators of a stromal-epithelial interaction.

**Fig 7:**
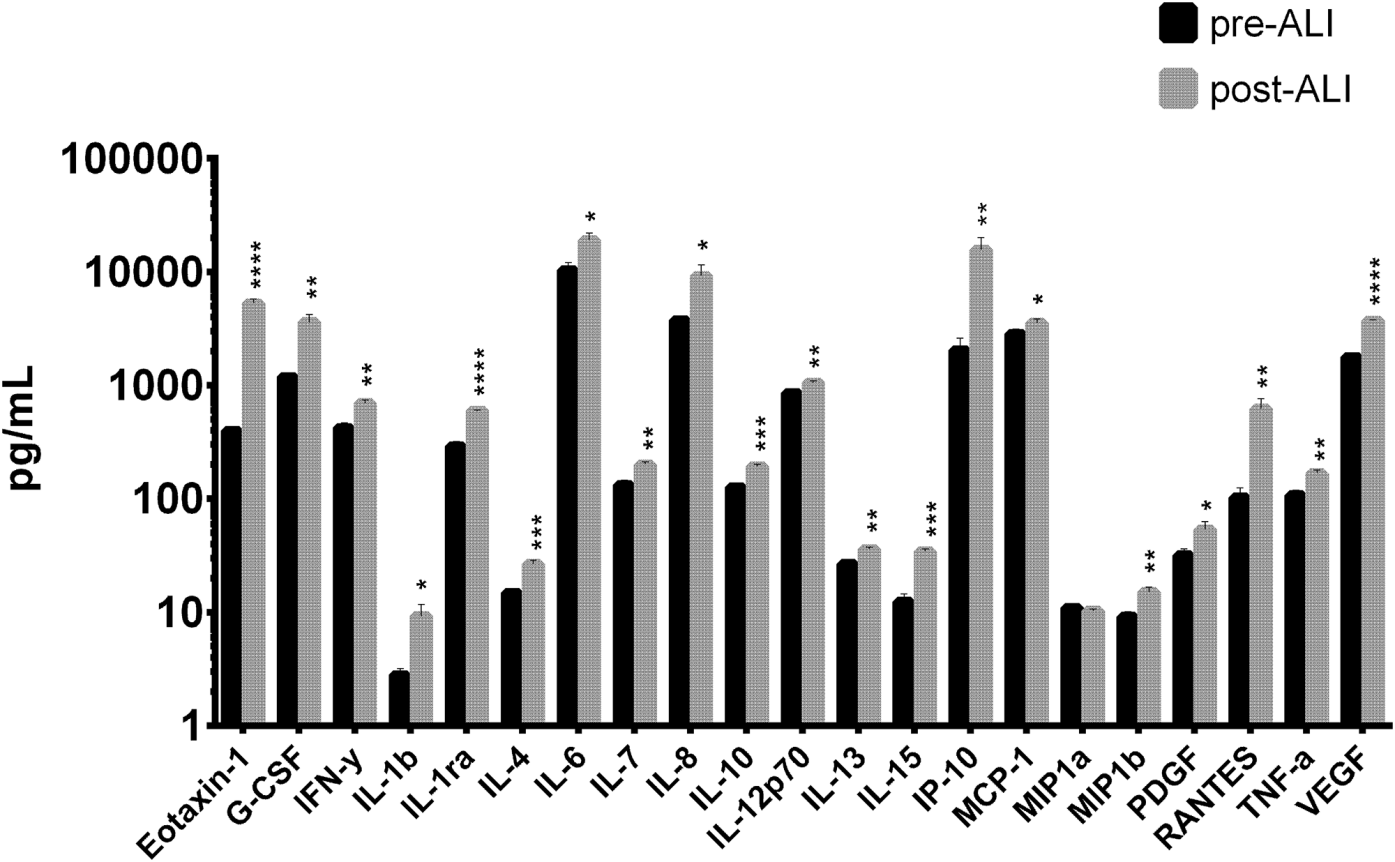
BEM cytokine secretion profile. Histogram plot representing cytokine concentration (pg/mL) in culture basal supernatants collected after 7 days from the model assembly before ALI transition (pre-ALI) and after 28 days from the model assembly at the end of the differentiation protocol (post-ALI). Data are represented as mean values (+ SD) of 3 biological replicates. Multiple t-test was performed to determine statistical significance. ****p<0.0001; ***p<0.001; **p<0.01; *p<0.05.

The majority of analyzed secreted factors augmented during the differentiation period (post-ALI) respect to pre-ALI conditions in the standard BEM configuration (Fig 7). In absolute values major secretion levels were identified for IL6, IP-10, MCP-1, IL8, eotaxin, G-CSF, IL-12p70, indicating a sustained chemo-attractive profile.

### BEM represents a suitable *in vitro* model to study bacterial adaptation to the respiratory tract

**Fig 8:**
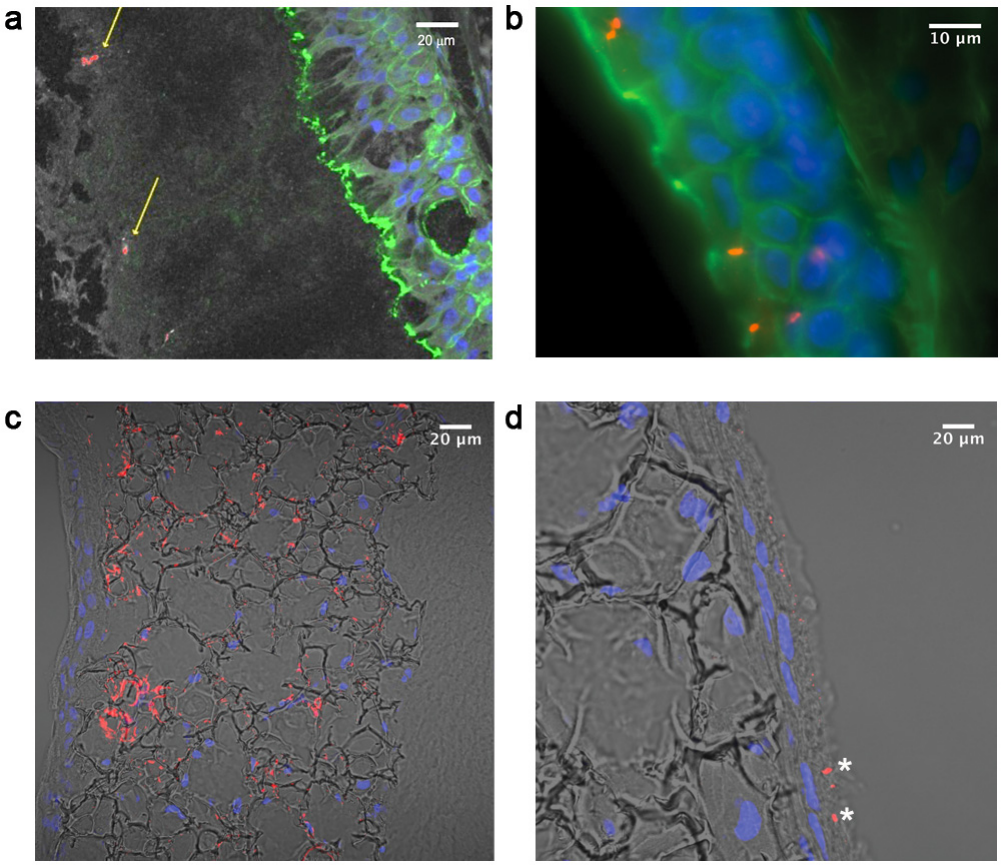
Immunofluorescence characterization of NTHi-infected BEM. a) Bacteria (red), revealed by using polyclonal antibodies against the whole bacterium, are embedded into a thick mucus layer (white). The contour of epithelial cells is delineated by phalloidin staining (green). b) Bacteria (red) associated with and traversing the epithelial layer (phalloidin, green; DNA, blue). c) NTHi bacteria (red) colonizing the stromal compartment (transmitted light, grey; DNA, blue). d) Outer Membrane Vesicles (red) detected within the bottom side of the BEM stromal compartment (transmitted light, grey; DNA, blue) and bacteria (red), highlighted by asterisks.

The ultimate goal of our study was the validation of BEM under conditions that would mimic the *in vivo* host-pathogen interplay occurring during the adaptation/infection of microbial organisms to the mucosal environment. As a model microorganism we chose NTHi, a human commensal bacterium that is part of the normal flora of the upper airways and for which are available *in vivo* evidences for the capacity to reside inside the bronchial epithelium. In addition to being commensal, NTHi is implicated in mucosal infections, including otitis media and in the exacerbation from chronic obstructive pulmonary disease [28]. NTHi strain Fi176, a clinical isolate strain derived from a case of otitis media [29, 30], was used to apically infect the BEM. After 40h of infection the pseudo-tissue was fixed and cryo-sectioned for microscopic analysis. As expected, NTHi was detected within the mucus layer that acts as the principal barrier to hinder bacterial contact with the epithelium (Fig 8a). According to pre-existing evidences of NTHi mucosal infection indicating that the bacterium is actively involved in crossing the epithelial barrier [31–33], we were able to detect bacteria infiltrating within the epithelial sheet (Fig 8b) or in the stromal compartment organized in macro-aggregates (Fig 8c). This phenotype resembled the initial stage of colonization of respiratory tissues by mucosal pathogens, which is characterized by the formation of stable communities contributing to the persistence of bacteria in the host. Importantly, numerous vesicles of bacterial origin (named OMVs, outer membrane vesicles) were identified at the bottom side of the stromal compartment (Fig 8d). This observation is in accordance with *in vivo* evidence suggesting that NTHi releases large amount of membrane-based vesicles during infection [34]. These data further support the importance of the model as a valid surrogate for studying microbial infections.

## Discussion

The scientific understanding of how microbes emerge, persist and adapt to the host is continuously growing. This result has been achieved thanks to a number of new technological applications that have greatly helped the deep comprehension of the mechanisms exploited by pathogens to benefit from humans. In this context, the *in vitro* reconstitution of human tissues by 3D cellular models represents a unique opportunity to overcome a number of issues related to the use of traditional cell lines or animal models far from representing the natural environment in which microorganisms usually reside. The relevance of this aspect, along with strong ethical concerns, is also driving governments and regulatory organizations to support the implementation of alternative methods to animal testing. The model proposed in this paper by attempting to rebuild the airway wall is expected to provide to the scientific community an *in vitro* tool suitable for the study of respiratory infections. Bacterial colonization of the human airway mucosa is a complex multiphasic process, involving different cellular subtypes. Indeed, to successfully persist in the mucosal epithelium, microbes would not only need to compete for nutrients with other resident bacteria but also to avoid mucociliary and immune clearance. An important limitation of the models currently used to study host-bacterial processes in the airways is that they do not provide a thick significant 3D structure, leading to the accumulation of growing bacteria within few layers of cells. This aspect limits the infecting time to few hours as the epithelium is heavily impacted as soon as bacteria form a critical mass. In our model this phenomenon is partially avoided by the fact that bacteria do not accumulate within the epithelial space as they have the opportunity to proceed through their colonization route by passing this intermediate barrier and forming large aggregates in the stromal compartment with no apparent damage of the surrounding tissue. The infiltration underneath the epithelial barrier of respiratory pathogens such as NTHi, *Streptococcus pneumoniae* and *Staphylococcus aureus* seems to be the mechanisms by which these bacteria are able to avoid immune system clearance and produce recurrent infections [35, 36]. According to this scenario, the formation of a physiological epithelial barrier is of extreme importance in order to study the mechanisms by which pathogens traverse the respiratory epithelium.

Our data indicate that a basal membrane forms in the BEM, constituted of a continuous laminin layer to which epithelial cells can anchor through the ITGA-6 component. By taking into account that under these conditions epithelial cells present in the BEM also develop sealing tight junctions, we postulate that our model could be successfully implemented for the study of epithelial barrier crossing by respiratory pathogens. A nice example of the exploitation of the model to study host-pathogen interaction was also reported in our study. Indeed, we monitored the infection of the BEM by a strain of NTHi and observed an invasive phenotype comparable to the one reported for bronchial and lung COPD explants [32, 37], in which bacteria actively transverse the epithelial barrier in order to reach the stromal compartment where they can better protect from antibodies and immune cell clearance [38]. Notably, the macroscopic morphological structure of the tissue seemed not to be influenced by NTHi since the epithelial sheet and the underlying compartment preserved their integrity also if proliferating bacteria were invading the whole tissue. We did not observe evident damage signals also in the epithelial sheet, confirming the innocuous status of the pathogen during its occupancy of the human respiratory tract of healthy individuals.

The fact that we spotted the presence of BCs and ClCs within the epithelium opens interesting perspective on the fact that these cells could play a role in repairing the tissue after damage induced by pathogens. Under the reported infection conditions, we also detected high amounts of OMVs within the BEM. These secreted lipid-based structures are known to be produced by NTHi during infection and to stimulate the release of anti-microbial peptides and immuno-modulatory molecules by epithelial cells [39, 40]. Interestingly, even though the presence of OMVs was associated with all the BEM, a clear enrichment of the vesicles was noted at the bottom level of the stromal compartment.

In conclusion, we think that the BEM can be a valuable tool to dissect long-term host-bacterial interplay opening new opportunities to design efficacious intervention strategies to treat fastidious pathogen-driven respiratory diseases.

## Materials and Methods

### Cell culture

Human normal lung fibroblasts (NHLFs) (Clonetics^™^) were expanded in FGM-2 (Lonza) medium and used for 10 passages. Normal human tracheobronchial epithelial cells (NHBE) (Clonetics^™^) were expanded at early passages and maintained in BEGM (Lonza). NHFLs and NHBE cells used in this study originate from two adults (1 male, 1 female) of age comprised between 43 and 62 years. PneumaCult^™^-ALI (STEMCELL Technologies^™^) was used during the last 3 weeks of differentiation in air liquid interface (ALI) conditions. StemPro Accutase (Gibco) solution was used to detach the cells during the passaging.

### 3D cell culture assembly

Alvetex inserts (Reinnervate) were pretreated and coated with Puramatrix (BD Falcon) (0,8 mg/mL) to support fibroblasts adhesion. 5′10^5^ NHLF cells were seeded and cultured in FGM2 medium inside Alvetex scaffolds for 3 days to allow the formation of a stromal tissue equivalent. Subsequently 180uL of 2 mg/mL type I rat tail collagen solution (Sigma) were loaded on top of the insert to obtain a coating support for epithelial cells. On day 5, NHBE cells (1′10^5^/cm^2^) were layered on the collagen sheet and cultured until confluence in BEGM medium (Lonza). From day 7 and for the next 3 weeks, the NHBE cells were cultured in PneumaCult-ALI (Stemcell Technologies) according to producer instructions to induce bronchial epithelial differentiation.

### Preparation of histological sections

The samples were fixed O/N in 4% buffered formaldehyde pH 7.6, cut in 2 equal halves along the sagittal plane and processed for paraffin embedding. 3μm sections were cut from the paraffin-embedded sample with Leica RM2255 microtome. Deparaffinized and re-hydrated histological sections were stained with Carazzi’s Hematoxylin and eosin and finally dehydrated. Images were acquired by using a Leica DM5000B microscope. For hematoxylin-eosin-alcian blue staining a first staining step was done for 30 min with Alcian Blue 8GS 1% pH 2.5 (Sigma) leaving the samples unwashed before fixation.

Samples previously fixed for at least 24 h as above were soaked O/N at 4°C in sucrose 15% and then in sucrose 30% bath before being included in O.C.T. Samples were then snap-frozen with liquid nitrogen-cooled isopentane and stored at -80°C until processed for cryosectioning. 10 μm or 20 μm thick sections were used for immunofluorescence labeling.

### Immunostaining

For immunofluorescent staining of the unsectioned BEM, intact samples were washed with PBS and fixed as above for 12 h. For mucin detection samples were alternatively fixed in cold Acetone/ Methanol solution for 10 minutes. Samples were then cut in different parts and washed twice in PBS. Fixed samples were also incubated in permeabilizing solution containing PBS 1% Triton X-100. Nonspecific binding was blocked incubating samples with PBS containing 10% goat serum, 3% BSA and 0,1% triton. Primary antibodies were diluted 1:250 in antibody dilution buffer (PBS 1% BSA) and left O/N at 4°C with gentle rocking. Subsequently samples were incubated for 1h at RT with Alexafluor-conjugated secondary antibodies (1:500) and phalloidin (1:100) (Molecular Probes). DNA was labeled with Hoechst 33342 (1:10000) or DAPI.

For the staining of cryosectioned BEM slices, samples were rehydrated with PBS and blocked for 30 minutes. After 1 wash in PBS 1% BSA, slides were incubated with primary antibodies diluted in PBS 0,1% Triton for 1 h RT. After 3 washes, samples were incubated with Alexafluor-conjugated secondary antibodies and phalloidin for 30 minutes. Finally, after 2 washes with PBS samples were counterstained with Hoechst 33342 and the slides were mounted using Antifade Reagent (Molecular Probes). Images were acquired by using an LSM710 confocal microscope (Zeiss).

Overlapping tiled images were acquired with an epifluorescent Zeiss Observer microscope and assembled by using the MosaiX module of AxioVision suite software (Zeiss).

For immunohistochemistry preparations, to preserve epitope structure paraffin embedded slides were pretreated with Cell Conditioning 1 solution (CC1) (Roche) incubated with polyclonal α-laminin antibodies for 12h and revealed with ChromoMap DAB kit (Roche). A list of the antibodies used in this study can be found in S1 Table.

### Electron Microscopy

Samples were divided and fixed O/N at 4°C in sodium cacodylate buffer 0,1M containing 2,5% glutaraldehyde and 2.5% formaldehyde and then post-fixed in 1% OsO4. Samples were then dried by the critical point method using CO^2^ in a Balzers Union CPD 020. For SEM they were sputter-coated with gold in a Balzers MED 010 unit. For TEM, the dried samples were embedded in LRWhite resin and stained with uranyl acetate and lead citrate.

### Cytokines Profiling

To characterize the cytokine secretion profile of the BEM, culture media were collected before and after the ALI period. Briefly, at day 5 differentiation medium was added to the BEM and collected at day 7 (pre-ALI condition). Regarding the post-ALI condition, BEM was maintained for 21 days in differentiation medium. During the post-ALI the medium was changed at intervals of 2 days and the cytokine profile measured at day 28 from initial assembling of BEM. For cytokine profiling, collected medium from the basal side of the well was centrifuged at 9500 g and stored at -80°C. Thawed undiluted media from biological triplicates were tested by Bio-Plex Pro^™^ Human Cytokine 27-plex (Bio-Rad), according to the producer protocol. BEGM and Pneumacult-ALI reference wells were used as basal controls.

### NTHi infection

NTHi Fi176 strain grown on agar-chocolate plates (Biomerieux) was inoculated in Brain Heart Infusion (BHI) medium supplemented with haemin and NAD until A_600_ = 0.4. Bacteria were harvested by centrifugation and resuspended in PneumaCult ALI maintenance medium, devoid of antibiotics. The apical side of the BEM was washed with warm PBS to reduce the mucus content and facilitate the binding of bacteria to the cells. 200 uL of bacterial suspension (2′10^7^ c.f.u., MOI 100:1) were added on the apical side of the BEM and incubated for 2 h at 37°C with 5% CO2. Non-adherent bacteria were removed by washing with PBS and treated BEMs were further incubated for 40h or 56h. Samples were finally fixed as above and cryosectioned for immunofluorescence analysis, bacteria were detected via anti-NTHI serum produced immunizing rabbits.

## Supporting Information

S1 Fig NHLFs deposit extra cellular matrix components within the porous scaffold after 1 week of culture. a) Immunofluorescence staining for fibronectin (blue is DNA, green is F-actin, red is fibronectin). b) Immunofluorescence staining for collagen (blue is DNA, green is F-actin, red is collagen type I); (a) and (b) represent maximum z-projection images of two separate stack acquisitions.
(TIF)

S2 Fig Comparison of the mucociliary phenotype between the triple co-culture model including MSCs and the standard BEM. a-b) Representative tiled images (n = 3) of an MSC-BEM and a standard BEM stained for DNA (blue), acetylated tubulin (red) and MUC5AC (green). Scale bar is 500 μm. c -d) Haematoxylin-eosin-alcian blue staining of a standard BEM and of an MSC-BEM, respectively (GCs are stained in blue).
(TIF)

S3 Fig Characterization of MoDCs in tryple co-cultures after 28 days. a) Immunofluorescence staining of MoDCs in mature fixed DC-BEM (blue is DNA, red is F-actin, green is CD45).
(TIF)

S4 Fig Cytokine accumulation in different BEM configurations. a) Differential cytokine concentrations in standard BEM and DC-BEM cultures. P-value calculated according to unpaired t-test (*, P<0.05; **, P < 0.01; ***, P < 0.001). b) Differential RANTES and IP-10 concentrations in standard BEM, MSC-BEM and DC-BEM. P**-**value calculated according to one-way anova test (*, P<0.05; ***, P < 0.001).
(TIF)

S1 File Appendix. (DOC)

S1 Table Commercial antibodies used in the study. (DOCX)
