# Supplementary figures and images for: 3D Reconstruction of the Human Airway Mucosa In Vitro as an Experimental Model to Study NTHi Infections
Source: PLoS One. 2016 Apr 21;11(4):e0153985. doi: 10.1371/journal.pone.0153985 (PMC4839639; doi:10.1371/journal.pone.0153985)

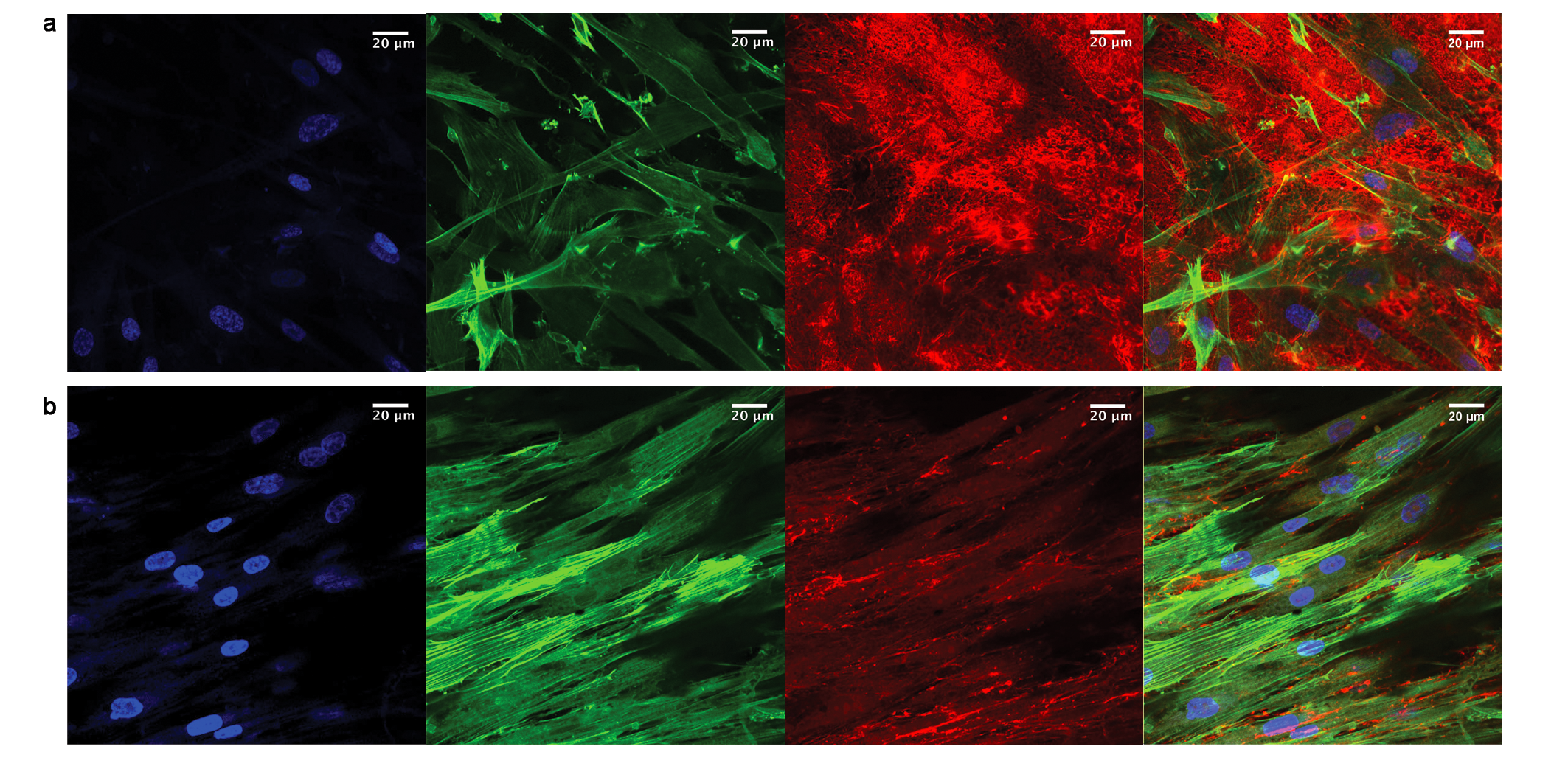

Supplement: S1 Fig — a) Immunofluorescence staining for fibronectin (blue is DNA, green is F-actin, red is fibronectin). b) Immunofluorescence staining for collagen (blue is DNA, green is F-actin, red is collagen type I); (a) and (b) represent maximum z-projection images of two separate stack acquisitions. (TIF) [file pone.0153985.s001.tif]

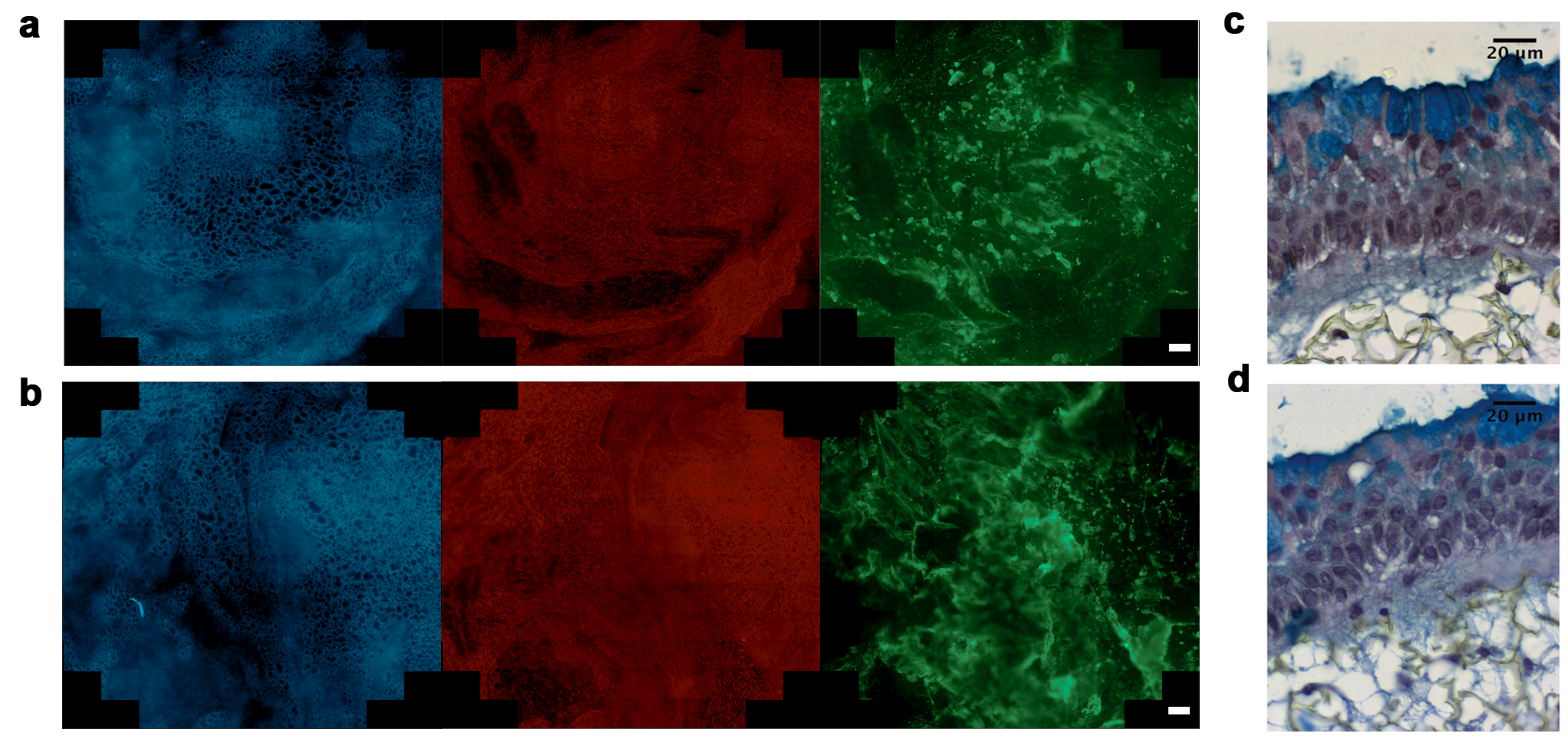

Supplement: S2 Fig — a-b) Representative tiled images (n = 3) of an MSC-BEM and a standard BEM stained for DNA (blue), acetylated tubulin (red) and MUC5AC (green). Scale bar is 500 μm. c -d) Haematoxylin-eosin-alcian blue staining of a standard BEM and of an MSC-BEM, respectively (GCs are stained in blue). (TIF) [file pone.0153985.s002.tif]

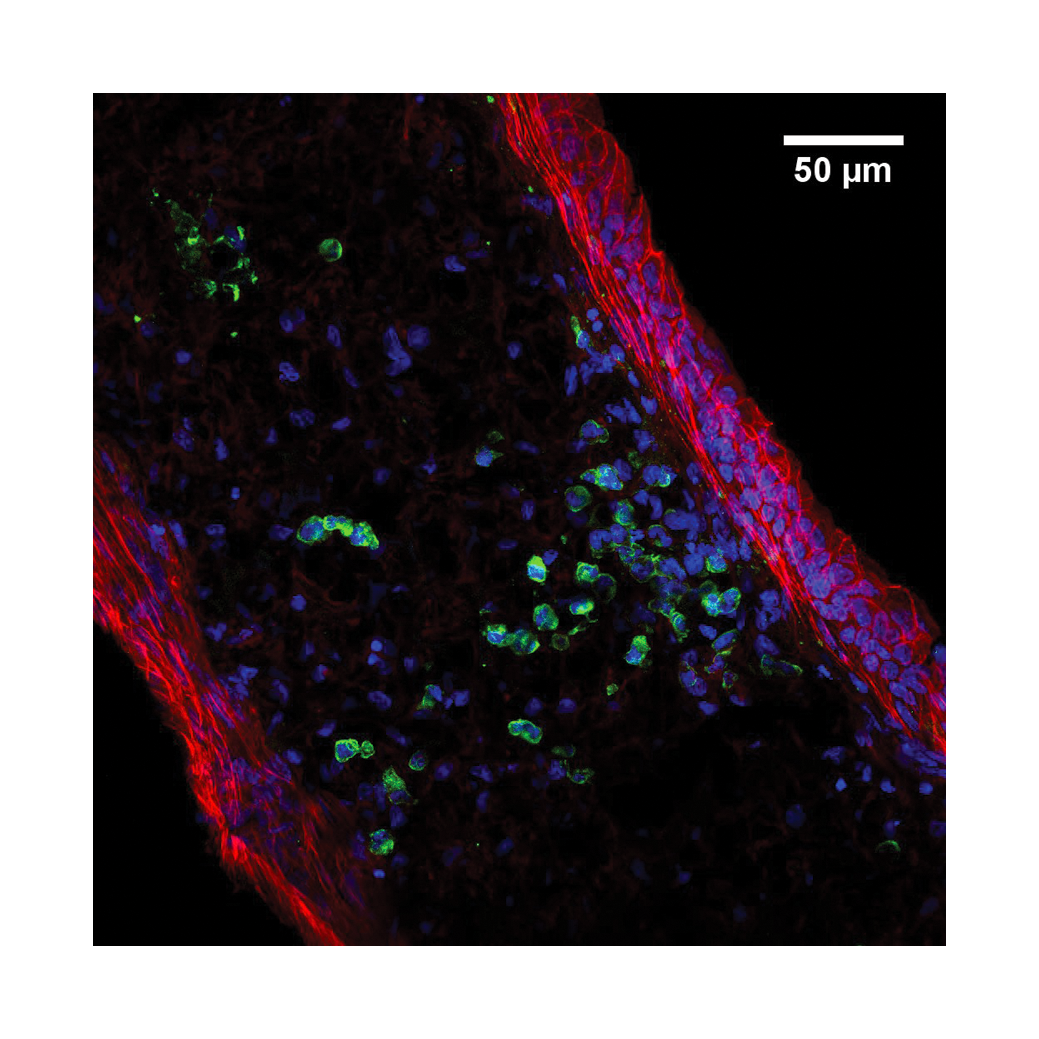

Supplement: S3 Fig — a) Immunofluorescence staining of MoDCs in mature fixed DC-BEM (blue is DNA, red is F-actin, green is CD45). (TIF) [file pone.0153985.s003.tif]

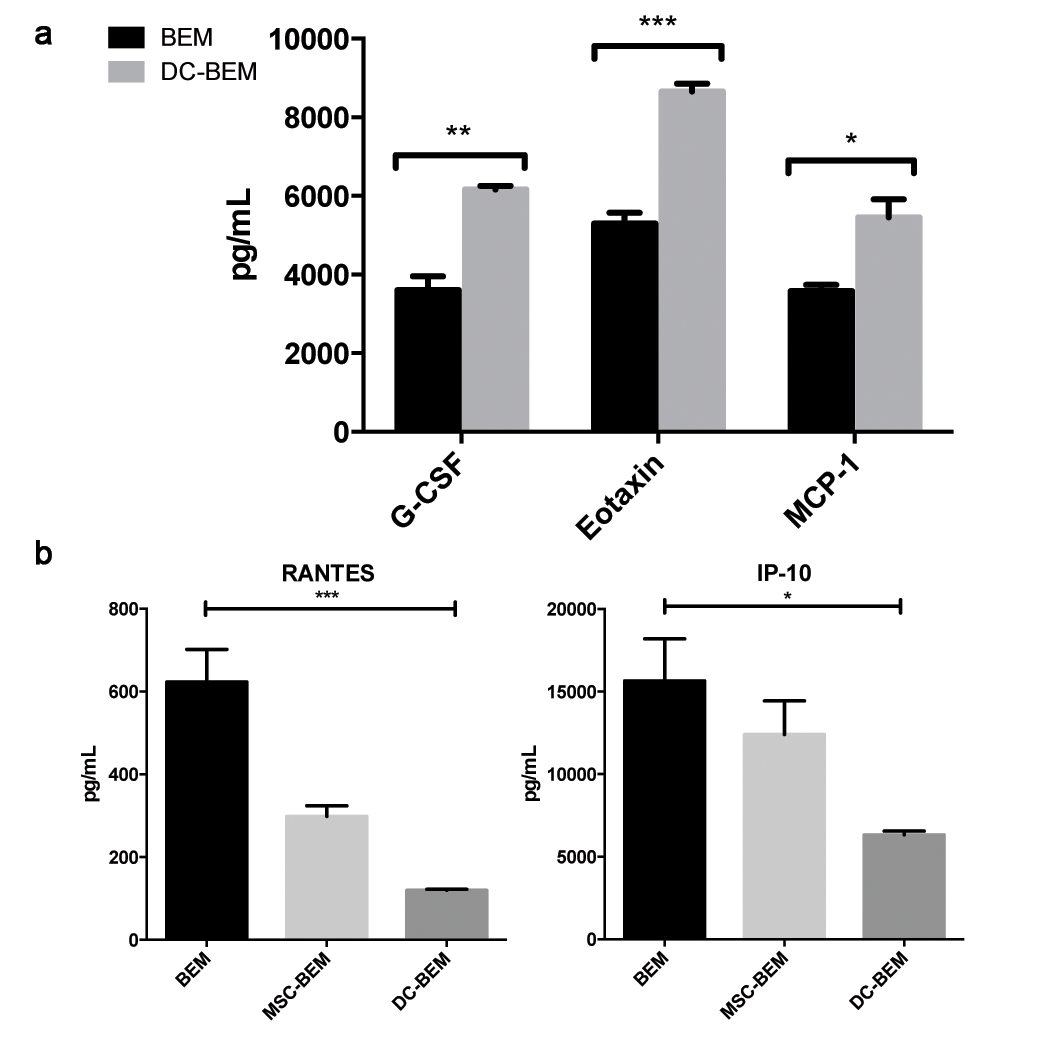

Supplement: S4 Fig — a) Differential cytokine concentrations in standard BEM and DC-BEM cultures. P-value calculated according to unpaired t-test (*, P<0.05; **, P < 0.01; ***, P < 0.001). b) Differential RANTES and IP-10 concentrations in standard BEM, MSC-BEM and DC-BEM. P-value calculated according to one-way anova test (*, P<0.05; ***, P < 0.001). (TIF) [file pone.0153985.s004.tif]
