# Supplementary material for: 3D Reconstruction of the Human Airway Mucosa In Vitro as an Experimental Model to Study NTHi Infections
Source: PLoS One. 2016 Apr 21;11(4):e0153985. doi: 10.1371/journal.pone.0153985 (PMC4839639; doi:10.1371/journal.pone.0153985)
Supplement: S1 File — (DOC) [file pone.0153985.s005.doc]

**Appendix I**

In order to increase the physiological complexity of the BEM, we decided to introduce a third cellular component to the system: immune cells or adult stromal stem cells. These cells are likely to enrich the tissue environment and reinforce the host defense equipment proper of normal human airways. The introduction in the model of Mo-DCs or MSCs did not apparently affect the overall phenotype obtained in the standard BEM (S2 Fig). Indeed, we successfully obtained a well-defined mucociliary layout also for DC-BEM and MSC-BEM variants (data not shown). According to the itinerant behavior of dendritic cells, we found that the majority of MoDCs were infiltrating within the stromal compartment while some continued to reside in the collagen layer (S3 Fig).

A number of cytokines such as G-CSF, eotaxin and MCP1 were more expressed in the DC-BEM (S4a Fig). The most intriguing result was the reduced accumulation of IP-10 and RANTES in DC-BEM compared to standard BEM (S4b Fig), which may suggest the entrapment of chemokines by dendritic cells through the variety of immune receptors present on their surface and known to be modulated during differentiation (Fischer *et al.*, Journal of immunology, 2001). A similar trend was observed when MSC were added to BEM, an effect that well correlates with their immune-modulatory properties (Bruno *et al*, Immunology letters, 2015)

**Matherials and Methods**

**Generation of dendritic cells**

PBMCs were isolated by Ficoll-Paque™ density gradient centrifugation from healthy donors buffy coats. PBMCs were then processed using MACS® Technology (Miltenyi Biotec) to obtain CD14+/ CD16+ monocytes by negative selection. Monocytes were seeded in 12-well plates at 5*10^5^/mL in advanced RPMI 1640 Medium (Gibco®) supplemented with 10% Fetal Bovine Serum, 50 uM beta-mercaptoethanol, 2mM GlutaMAX™ and antibiotics. To promote in vitro differentiation into immature Monocyte-derived Dendritic Cells (MoDC), purified monocytes were cultured for 6 days in the presence of 50 ng/mL of human recombinant GM-CSF and IL-4 (Gibco®). Cytokines supplemented medium was refreshed once after 3 days. On 7th day cells were harvested and stained with antibody cocktails (Miltenyi) and surface cell phenotype was analyzed by flow cytometry to evaluate the differentiation stage. MoDC preparations with concurrent expression of CD209 and CD1a higher than 80% and with downregulation of CD14 were used in the assembly of the DC-BEM.

**Mesenchymal stromal cell (MSC) culture**

Umbilical Cord-Derived Mesenchymal Stem Cells (UC-MSC) (ATCC) screened for specific stem cell surface antigens and derived from human Wharton’s Jelly were expanded in MesenPRO RS™ (Gibco®) and passaged using accutase solution.

**Triple co-culture BEM**

Triple co-culture bronchial equivalent models were assembled as above with small modifications: UC-MSC cells (ATCC) were included within the scaffold (UC-MSC/NHLF co-culture density ratio was 1:2) to obtain the MSC-BEM while Mo-DCs were included in the collagen suspension used to separate the epithelial sheet from the scaffold (defined DC-BEM).
