# Supplementary material for: 3D Reconstruction of the Human Airway Mucosa In Vitro as an Experimental Model to Study NTHi Infections
Source: PLoS One. 2016 Apr 21;11(4):e0153985. doi: 10.1371/journal.pone.0153985 (PMC4839639; doi:10.1371/journal.pone.0153985)
Supplement: S1 Table — (DOCX) [file pone.0153985.s006.docx]

| Name | Code |
| --- | --- |
| α -β tubulin IV | T7941 Sigma |
| α-Laminin | T9393 Sigma |
| α-collagen I | Ab34710 Abcam |
| α- MUC5AC | MAB2011 Millipore |
| α-SCGB1A1 | SAB2102083 Sigma |
| α-CK5 | MAB3224 Millipore |
| α-ZO1 | 40-2200 Life Technologies |
| UltraMap anti rabbit HRP | 760-4315 Roche |
| α-NGFR | Ab8874 Abcam |
| PE- α- CD1a | 12-0019 eBioscience |
| MODCdifferentiation inspector | 130-093-567 Miltenyi |
| α-p63 | ab735 Abcam |
| α-ITGα6 | ab20142 Abcam |
| α-CD45 | MHCD4501 Life technologies |
